# Efficacy and safety of CKD-495 in acute and chronic gastritis: A Phase III superiority clinical trial

**DOI:** 10.1097/MD.0000000000035926

**Published:** 2023-12-08

**Authors:** Seung Young Seo, Soo Teik Lee, Sung Kook Kim, Hoon Jai Chun, Geun Am Song, Dong Ho Lee, Jae Jun Kim, Jin Il Kim, Young Chan Lee, Tae Nyeun Kim, Sam Ryong Jee, Seon-Young Park, Jae Gyu Kim, Jong-Jae Park, Sang Gyun Kim, Jae Myung Park, Jung Ho Park, Shin Jung Park, Oh Young Lee

**Affiliations:** a Department of Internal Medicine, Research Institute of Clinical Medicine of Jeonbuk National University-Biomedical Research Institute of Jeonbuk National University Hospital, Jeonju, Korea; b Department of Internal Medicine, Kyungpook National University Hospital, Kyungpook National University School of Medicine, Daegu, Korea; c Department of Internal Medicine, Korea University College of Medicine Anam Hospital, Seoul, Korea; d Department of Internal Medicine, Pusan National University Hospital, Pusan National University School of Medicine, Busan, Korea; e Department of Internal Medicine, Seoul National University Bundang Hospital, Seoungnam Korea; f Department of Medicine, Samsung Medical Center, Sungkyunkwan University School of Medicine, Seoul, Korea; g Department of Internal Medicine, College of Medicine, The Catholic University of Korea, Yeouido St. Mary’s Hospital, Seoul, Korea; h Department of Internal Medicine, Severance Hospital, Yonsei University College of Medicine, Seoul, Korea; i Department of Internal Medicine, Yeungnam University College of Medicine, Daegu, Korea; j Department of Internal Medicine, Inje University Busan Paik Hospital, Inje University College of Medicine, Busan, Korea; k Department of Internal Medicine, Chonnam National University Medical School, Gwangju, Korea; l Department of Internal Medicine, Chung-Ang University College of Medicine, Seoul, Korea; m Department of Internal Medicine, Korea University College of Medicine Guro Hospital, Seoul, Korea; n Department of Internal Medicine, Seoul National University Hospital, Seoul, Korea; o Department of Internal Medicine, Seoul St. Mary’s Hospital, College of Medicine, The Catholic University of Korea, Seoul, Korea; p Department of Internal Medicine, Kangbuk Samsung Hospital, Sungkyunkwan University School of Medicine, Seoul, Korea; q Chong Kun Dang Research Institute, Chong Kun Dang Pharmaceutical Corporation, Seoul, Korea; r Department of Internal Medicine, Hanyang University College of Medicine, Seoul, Korea.

**Keywords:** acute gastritis, chronic gastritis, *Cinnamomum cassia*, erosion, mucoprotective agent

## Abstract

**Background:**

Despite the availability of numerous treatment options, many patients with gastritis experience only partial symptom relief. CKD-495, a newly developed product with the active ingredient extracted from *Cinnamomum cassia* Presl., has demonstrated anti-inflammatory and antioxidant activity in vitro and an in vivo protective effect against gastric damage by stimulating mucus secretion. This study compared the efficacy and safety of CKD-495 with *Artemisiae argyi* folium (AAF) for the treatment of acute and chronic gastritis. AAF, a gastric mucosa protective agent that promotes gastric mucosa regeneration, has been used clinically for about 20 years.

**Methods:**

This phase III multicenter, randomized, double-blind, parallel-group trial (ClinicalTrials.gov; NCT04255589) assigned 242 patients with endoscopically-proven gastric mucosal erosions to receive CKD-495 75 mg (n = 122) or AAF 60 mg (n = 120), respectively, with placebo (for double-blind purposes) 3 times a day for 2 weeks. The primary efficacy endpoint was the erosion improvement rate. Secondary endpoints included erosion cure rates, and improvement rates for edema, redness, hemorrhage, and gastrointestinal (GI) symptoms. Drug-related adverse events were evaluated.

**Results:**

The erosion improvement rate was significantly higher in the CKD-495 group than in the AAF group for both the full analysis set (55.9% vs 39.4%, *P *= .0063) and per-protocol set (54.6% vs 38.2%, *P *= .0084). In addition, the erosion improvement rate in patients with acute or chronic gastritis showed that the CKD-495 group had better improvement of erosion than the AAF group, especially in patients with chronic gastritis. Analysis of secondary endpoints, which included erosion cure rate and the improvement rates of edema, redness, hemorrhage, and GI symptoms, showed that the CKD-495 group was more effective than the AAF group. There were no significant between-group differences in safety profiles. No serious adverse events or adverse drug reactions occurred.

**Conclusions:**

These results demonstrate that CKD-495 75 mg is superior to AAF 60 mg in terms of the endoscopic improvement rate of erosions in patients with acute or chronic gastritis. This new mucoprotective agent, CKD-495, can be considered the therapy of choice for symptomatic relief and healing of gastritis.

## 1. Introduction

Gastritis is defined as inflammation of the gastric mucosa which is confirmed by histological evaluation. It is a heterogeneous pathological condition and is one of the most common diseases to need medical consultation in Korea. Inflammation of the gastric mucosa results from an imbalance between mucosal defensive and aggressive factors such as gastric acidity, mucus-bicarbonate barrier, and gastric hormones (i.e., gastrin, pepsinogens I, II).^[1,2]^ The impaired mucosal defensive mechanism can cause mucosal injury which can result in gastritis or gastric ulcer even in children.^[3,4]^ Acute gastritis refers to a state of acute inflammation in the gastric mucosa, redness, edema, and infiltration of acute cells. On the other hand, chronic gastritis is the infiltration of chronic inflammatory cells into the gastric mucosa and is accompanied by gastric atrophy and intestinal metaplasia. Gastritis can be classified into acute or chronic forms based upon the Sydney System, and chronic gastritis can be sub-classified as nonatrophic, atrophic, and special types.^[5,6]^ Another classification, based on the etiology of gastritis, can be used when considering the potential role of various causes in the progression to gastric mucosal atrophy.^[7]^

The current treatment of gastritis is based mainly on controlling gastritis-associated symptoms, such as epigastric pain, nausea/vomiting, and abdominal distention, by using acid suppressive agents, gastrointestinal (GI) motility modulating agents, antacids or gastric mucoprotective agents.^[8]^ Gastric mucoprotective agents are used in combination with acid-suppressing agents such as a proton pump inhibitor (PPI) or H_2_-receptor antagonist (H2RA) or alone. Despite these treatment options, many patients with gastritis experience only partial symptom relief.

CKD-495, a product derived from *Cinnamomum cassia* Presl., has been reported to have anti-inflammatory^[9,10]^ activities and antioxidant activity in vitro and a protective effect against gastric damage in vivo by stimulating mucus secretion. Four compounds (4-hydroxycinnamaldehyde, 3-4-dihydroxybenzaldehyde, trans-ferulic acid, and cinnamic acid) were found to be significant in CKD-495. As a gastric mucosa protective agent, CKD-495 and 4-hydroxycinnamaldehyde shows excellent anti-inflammatory effect by suppressing nuclear factor-κB (NF-κB) and mitogen-activated protein kinase signal transduction.^[11–13]^ It also increases the biosynthesis of the cytoprotective substance prostaglandin E_2_, promotes the secretion of gastric mucus, which is a protective factor of the gastric mucosa, and increases the amount of glutathione, which exhibits an antioxidant detoxification mechanism. CKD-495 is a product with enhanced defense and recovery functions of the stomach itself, such as suppressing myeloperoxidase, a marker of neutrophil inflammation.^[11–13]^

In a previous phase II trial to assess the optimal dose and safety of CKD-495 for the treatment of gastritis (NCT03437785), CKD-495 75 mg demonstrated efficacy and safety for improving erosion in patients with acute or chronic gastritis (Chong Kun Dang Pharmaceutical Corp., data on file). The aim of this study was to compare the efficacy and safety of CKD-495 75 mg versus *Artemisiae argyi* folium 95% ethanol ext.(20→1) (AAF [an ethanol extract of *Artemisia asiatica* that is used for the treatment of gastritis]) 60 mg for the treatment of acute and chronic gastritis.

## 2. Methods

This phase 3 study was a multicenter, randomized, double-blind, parallel-group, phase III clinical trial to evaluate the efficacy and safety of CKD-495 in patients with acute or chronic gastritis. The study was conducted at 17 Korean hospitals from January 2020 to May 2021. The study protocol was reviewed and approved by the institutional review board of each hospital according to the ethical principles of the Declaration of Helsinki and Good.

Clinical Practice (Supplementary Data, Table S1, http://links.lww.com/MD/K957). All participants agreed to sign the informed consent form before enrollment. The study was registered at ClinicalTrials.gov (NCT04255589).

### 2.1. Patients

Subjects who participated in the clinical study underwent blood tests, urinalysis, and esophagogastroduodenoscopy (EGD) screening tests. Inclusion criteria were: male and female patients aged ≥ 19 years with acute or chronic gastritis and one or more gastric erosion on baseline EGD and with gastrointestinal symptoms at the time of enrollment or within 7 days. The acute and chronic gastritis were determined based on the investigator judgement, and criteria such as patient symptoms or disease duration were not set. Exclusion criteria were: patients who were unavailable for EGD; patients with a history of peptic ulcer or reflux esophagitis; patients who had undergone gastrointestinal surgery, such as surgery to inhibit gastric acid secretion or esophagogastric surgery (except simple closure of peptic ulcer perforation); patients with a history of GI malignancy or Zollinger-Ellison syndrome; patients with a history of thrombotic disorder (cerebral infarction, myocardial infarction, thrombophlebitis) or coagulation disorder; patients who had used anti-thrombotic agents such as warfarin; patients with abnormal aspartate aminotransferase or alanine aminotransferase (>2 times the upper limit of normal [ULN]); patients with abnormal serum creatinine (>1.5 times ULN); patients who took any of the following drugs within 2 weeks before enrollment (H2RAs, PPIs, antacids, potassium competitive acid blockers, prokinetics, prostaglandin analogs, gastric mucosal protective agents, corticosteroids, nonsteroidal anti-inflammatory drugs, aspirin); known hypersensitivities to the study drug; genetic disorders such as galactose intolerance, Lapp lactase deficiency, or glucose-galactose malabsorption; any other clinical trial medications within 3 months before enrollment; pregnant or lactating patients; fertile women who did not consent to using medically-permitted contraceptive methods (condoms, diaphragms, oral contraceptives, injectable contraceptives, or intrauterine devices), or those with any other conditions or diseases that were regarded unsuitable by the investigator. Subjects who met the inclusion criteria were subsequently randomized to 2 treatment groups. Participants in each group received CKD-495 75 mg or AAF 60 mg (Stillen^®^; Dong-A Pharmaceutical Co., Ltd) with placebo (for double-blind purposes) 3 times a day for 2 weeks. Patients visited a study center for follow-up endoscopy 2 weeks after beginning the medication. Compliance was determined by the number of remaining tablets per drug type at the follow-up visit. Data for patients with ≥ 80% and ≤ 120% drug compliance were included in the per-protocol outcome measurements. The average duration of acute gastritis was 0.36 and 0.29 years in the CKD-495 and AAF groups, respectively. The average duration of chronic gastritis was 2.56 and 2.44 years in the CKD-495 and AAF groups, respectively.

### 2.2. Assessments

All patients were assessed by EGD, at baseline and 2 weeks after beginning the study drugs, for the evaluation of gastric erosion, edema, redness and hemorrhage. An erosion is a defect that does not extend beyond the lamina muscularis mucosae and in this study, all cases confirmed to be erosions at the investigator discretion were included. Gastric erosion was scored from 1 to 4 according to the number of erosions (1: no visible erosion, 2: 1–2 erosions, 3: 3–5 erosions, 4: ≥6 erosions). All endoscopic findings were evaluated by the investigators of each center first and then reviewed by a central reading center, which is composed of 3 independent endoscopy experts, to achieve a unified result. In addition, subjective GI symptoms such as epigastric pain (postprandial, fasting, nocturnal, regardless of diet), nausea/vomiting, abdominal distention, anorexia, heartburn and belching, were recorded at the beginning and end of treatment.

The primary efficacy endpoint was the improvement rate of erosions, defined as the percentage of patients with ≥ 50% decreased erosion. Patients who had ≥ 50% reduction in initial scores at the follow-up EGD 2 weeks after treatment initiation were classified as effective cases; effective (4 to 1 or 3 to 1 or 2 to 1 or 4 to 2), ineffective (else other). Secondary efficacy endpoints were erosion cure rates, and improvement rates of edema, redness, hemorrhage, and GI symptoms. Cure of erosions was defined as the disappearance of all erosions. Improvement rates of edema, redness, and hemorrhage were defined as a ≥ 50% reduction in the initial scores at the follow-up EGD 2 weeks after treatment initiation. Edema was scored 1 or 2, redness from 1 to 4, and hemorrhage was scored from 1 to 5 (Table 1). The improvement rate of GI symptoms was defined as a ≥ 50% reduction of the initial GI symptom scores. GI symptoms were scored from 0 to 3 according to the frequency of each symptom (0: absent, 1: once a week, 2: more than 2 times a week, and 3: daily). Symptom scores were obtained by the sum of the scores, with a maximum score of 18. Safety assessments included adverse events (AEs) and adverse drug reactions (ADRs), including any GI symptoms reported by patients and abnormalities in laboratory tests or physical examination. The safety set consisted of all subjects who took the study drug at least once after randomization.

**Table 1:** Endoscopic scoring of gastric erosion, edema, redness, and hemorrhage.

	Score	Findings
Erosion	1	None
	2	1–2 Erosions
	3	3–5 erosions
	4	≥6 of erosions
Edema	1	None
	2	Pale or whiter and slightly accentuated hexagonal area gastric pattern
Redness	1	None
	2	Minimal but obvious change
	3	Conspicuous patchy discoloration
	4	Color change is beefy-red in intensity
Hemorrhage	1	None
	2	1 Hemorrhagic lesion
	3	2–5 Hemorrhagic lesions
	4	6–10 Hemorrhagic lesions
	5	≥11 Hemorrhagic lesions or large area of confluent hemorrhage

### 2.3. Statistical analysis

This study aimed to demonstrate that the endoscopic improvement rate of CKD-495 75 mg, at Week 2 is superior to that of the AAF 60 mg, in patients with acute and chronic gastritis. To calculate the sample size, the improvement rate of erosion of CKD-495 and AAF was assumed based on the result of the Phase II study of CKD-495. When the improvement rate of CKD-495 75 mg and AAF 60 mg was set to 73.08% and 52.08%, respectively, the sample size calculated with a 2-sided 5% significance level (α), power (1- β) of 90%, dropout rate of 10%, and allocation ratio of 1:1 was 119 subjects per group, totally 238 subjects.

The analysis group was divided into 3 groups: full analysis set (FAS), per protocol analysis set (PPS) and safety set. The FAS included all randomized patients who received at least one dose of the study drug and underwent at least one efficacy measurement after treatment with the study drug. The PPS included all randomized patients except those who had major protocol violations or had poor drug compliance (<80% or >120%) and terminated the clinical trial ahead of schedule. The safety set included all patients who took the study drugs at least once. All efficacy endpoints were analyzed in the FAS and PPS.

In the analysis of baseline characteristics, continuous variables were presented as mean and standard deviation and were analyzed by the 2-sample t-test or Wilcoxon rank-sum test for between-group comparisons. Categorical variables were presented as number and proportion and were analyzed using chi-square or Fisher exact test for between-group comparisons. Endoscopic improvement rates of erosion were analyzed by the Wald test including study site as a covariate, and results are presented as odds ratio (OR), 95% confidence interval (95% CI) and *P* value. Comparisons between treatment groups in the analysis of secondary efficacy endpoints were reported as ORs and their 95% CIs by the Wald test, using study site as a covariate. Data were considered to be significant when *P* < .05. Statistical analyses were conducted using SAS version 9.4 (SAS Institute, Cary, NC, USA).

## 3. Results

**Figure 1.**
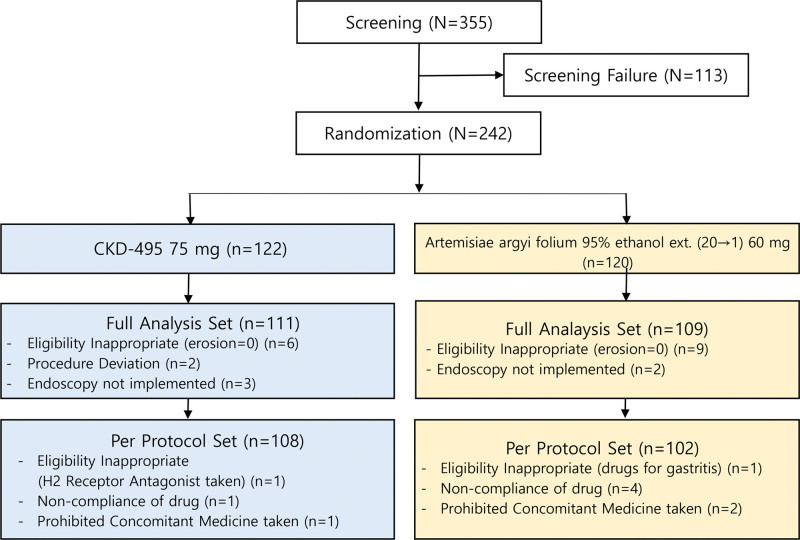
A Flow Patient Diagram according to the consort format.

A total of 355 patients were screened at 17 tertiary hospitals in Korea from January 2020 to May 2021. After excluding 113 patients during screening, 242 were randomly assigned to a treatment group (Fig. 1). A total of 220 patients were included in the FAS and 210 in the PPS. The study treatment groups were comparable with regard to demographics and disease-specific characteristics. There were no differences between the 2 groups in terms of sex, age, height, weight. The baseline endoscopic findings (erosion, edema, redness, and hemorrhage) and GI symptom score of patients were also comparable between the 2 groups (Table 2). The Safety set consisted of all 242 patients who took the study drug at least once after randomization. The number of patients in each analysis group is detailed in Table 3.

**Table 2:** Baseline characteristics of patients (n, % or mean ± SD).

Characteristics		CKD-495 75 mg (n = 111)	*Artemisiae argyi* folium 95% ethanol ext.(20→1) 60 mg (n = 109)	*P* value
Sex	male	40 (36.04)	45 (41.28)	.4241^C^
	female	71 (63.96)	64 (58.72)	
Age		48.4 ± 12.9	47.7 ± 13.5	.8272^W^
Height (cm)		163.92 ± 8.18	164.76 ± 7.83	.3168^W^
Weight (kg)		64.58 ± 13.04	64.52 ± 13.22	.8712^W^
Classification of gastritis	Acute gastritis	58 (52.25)	61 (55.96)	.5808^C^
	Chronic gastritis	53 (47.75)	48 (44.04)	
Duration of gastritis	Acute gastritis	0.36 ± 2.59	0.29 ± 1.92	.2008^W^
	Chronic gastritis	2.56 ± 3.73	2.44 ± 4.82	.4711^W^
Endoscopic findings				
Erosion	Grade 1	-	-	.9925^C^
	Grade 2	61 (54.95)	59 (54.13)	
	Grade 3	26 (23.42)	26 (23.85)	
	Grade 4	24 (21.62)	24 (22.02)	
Edema	Grade 1	82 (73.87)	81 (74.31)	.9409^C^
	Grade 2	29 (26.13)	28 (25.69)	
Redness	Grade 1	56 (50.45)	48 (44.04)	.1291^C^
	Grade 2	31 (27.93)	42 (38.53)	
	Grade 3	13 (11.71)	15 (13.76)	
	Grade 4	11 (9.91)	4 (3.67)	
Hemorrhage	Grade 1	74 (66.67)	74 (67.89)	.9440^F^
	Grade 2	16 (14.41)	13 (11.93)	
	Grade 3	16 (14.41)	17 (15.60)	
	Grade 4	3 (2.70)	4 (3.67)	
	Grade 5	2 (1.80)	1 (0.92)	
GI symptom score		5.50 ± 3.50	5.55 ± 3.14	.5869^W^

C : Chi-square test,

F : Fisher exact test,

W : Wilcoxon Rank Sum Test.

**Table 3:** Number of patients in the FAS, PPS and safety analyses.

	Number of patients		
	CKD-495 75 mg	*Artemisiae argyi* folium 95% ethanol ext.(20→1) 60 mg	Total
FAS analysis	111	109	220
PPS analysis	108	102	210
Safety analysis	122	120	242

FAS = full analysis set, PPS = per protocol analysis set.

### 3.1. Primary efficacy assessment

In the FAS analysis, patients in the CKD-495 75 mg group had a higher improvement rate of erosion than those in the AAF 60 mg group. (55.9% vs 39.4%, *P *= .0063; OR, 2.25; 95% CI, 1.26–4.03). Similar erosion improvement rates were observed in the PPS analysis (54.6% vs 38.2%, *P* = .0084; OR, 2.23; 95% CI, 1.23–4.05) (Table 4).

**Table 4:** Improvement rate of erosion in the FAS and PPS analyses (n, %).

Erosion improvement	CKD-495 75 mg	*Artemisiae argyi* folium 95% ethanol ext.(20→1) 60 mg	OR (95% CI)	*P* value
FAS	n = 111	n = 109		
Effective	62 (55.86)	43 (39.45)	2.25 (1.26–4.03)	.0063
Ineffective	49 (44.14)	66 (60.55)		
PPS	n = 108	n = 102		
Effective	59 (54.63)	39 (38.24)	2.23 (1.23–4.05)	.0084
Ineffective	49 (45.37)	63 (61.76)		

Results of the Wald test are presented as odds ratio, 95% confidence interval and *P* value (Covariate: Site).

FAS = full analysis set, PPS = per protocol analysis set.

In addition, we performed an analysis of the erosion improvement rate for patients in the FAS with acute or chronic gastritis. Patients in the CKD-495 75 mg group had better improvement of erosion than those in the AAF 60 mg group. In acute gastritis patients, the CKD-495 75 mg group had a numerically higher improvement rate of erosion compared with the AAF 60 mg group (62.1% vs 47.5%, *P *= .0531; OR, 2.24; 95% CI, 0.99–5.06). Furthermore, in chronic gastritis patients, the erosion improvement rate was significantly higher in the CKD-495 75 mg group than in the AAF 60 mg group (49.1% vs 29.2%, *P *= .0129; OR, 3.54; 95% CI, 1.31–9.59) (Table 5).

**Table 5:** Improvement rate of erosion in acute and chronic gastritis patients (n, %).

Erosion improvement	CKD-495 75 mg	*Artemisiae argyi* folium 95% ethanol ext.(20→1) 60 mg	OR (95% CI)	*P* value
Acute gastritis	n = 58	n = 61		
Effective	36 (62.07)	29 (47.54)	2.24 (0.99–5.06)	.0531
Ineffective	22 (37.93)	32 (52.46)		
Chronic gastritis	n = 53	n = 48		
Effective	26 (49.06)	14 (29.17)	3.54 (1.31–9.59)	.0129
Ineffective	27 (50.94)	34 (70.83)		

Results of the Wald test are presented as odds ratio, 95% confidence interval and *P* value (Covariate: Site).

### 3.2. Secondary efficacy assessment

**Figure 2.**
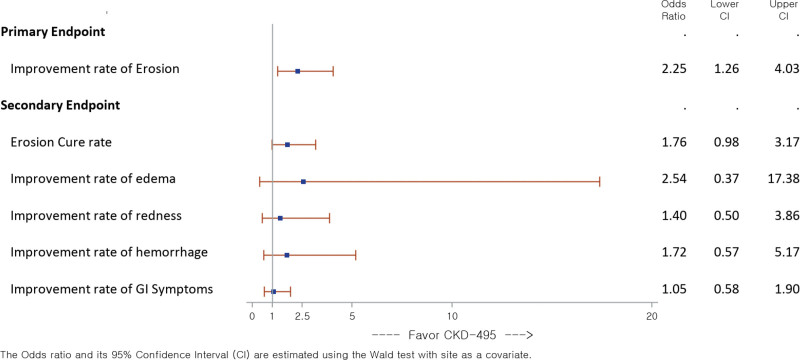
Primary and Secondary Efficacy endpoints results (ORs of the Wald test) (Full Analysis Set). OR = odds ratio.

Based on the FAS analysis, the results (ORs) of the Wald test showed that patients in the CKD-495 75 mg group were more likely to have improvement than those in the AAF 60 mg group for erosion cure rate (OR, 1.76; 95% CI, 0.98–3.17), and improvement rates of edema (OR, 2.54; 95% CI, 0.37–17.38), redness (OR, 1.40; 95% CI, 0.50–3.86), and hemorrhage (OR, 1.72; 95% CI, 0.57–5.17) and self-reported GI symptoms (OR, 1.05; 95% CI, 0.58–1.90) (Fig. 2). Similar findings were observed in the PPS analysis (data not shown).

### 3.3. Safety

No serious AEs or serious ADRs were reported in either treatment group during the study period. The incidence of AEs in the CKD-495 75 mg and AAF 60 mg groups was 3.28% (4 of 122) and 4.17% (5 of 120), respectively. All ADRs were mild with no significant differences between the 2 treatment groups (Table 6).

**Table 6:** Incidence of adverse events during the study (n, %).

Category	CKD-495 75 mg (n = 122)	*Artemisiae argyi* folium 95% ethanol ext.(20→1) 60 mg (n = 120)
Gastrointestinal		
Diarrhea	1 (0.82)	2 (1.67)
Large intestinal polyp	1 (0.82)	1 (0.83)
Gastroesophageal reflux	1 (0.82)	-
General		
Chills	1 (0.82)	-
Generalized edema	-	1 (0.83)
Infections and infestations		
coronavirus infection	1 (0.82)	-
Nervous system		
Headache	1 (0.82)	-
Laboratory		
Alanine aminotransferase elevation	-	1 (0.83)
Total	4 (3.28)	5 (4.17)

## 4. Discussion

The aim of this study was to assess the efficacy and safety of CKD-495 75 mg versus AAF 60 mg for the treatment of acute and chronic gastritis. The results of our phase III, multicenter, randomized, double-blind, parallel-group trial showed that CKD-495 75 mg was associated with a significantly better improvement rate of erosion, assessed endoscopically, than AAF 60 mg. In the current study, patients received treatment for 2 weeks, which is sufficient period to confirm the clinical utility of the findings to be accepted by regulatory authorities.^[14–18]^ Nevertheless, the 2-week treatment duration allowed us to demonstrate the superiority of CKD-495 75 mg over AAF 60 mg. In addition, analysis of the erosion improvement rate for patients with acute or chronic gastritis showed that patients in the CKD-495 75 mg group had better improvement of erosion than those in the AAF 60 mg group, especially for patients with chronic gastritis. Also, analysis of secondary endpoints (erosion cure rate and the improvement rates of edema, redness, hemorrhage, and GI symptoms) showed that patients in the CKD-495 75 mg group were more effective than those in the AAF 60 mg group.

No serious AEs or serious ADRs were reported in either group. AEs were observed in 3.28% of patients (4 of 122) in the CKD-495 75 mg group and 4.17% of patients (5 of 120) in the AAF 60 mg group, respectively. Diarrhea, gastroesophageal reflux, chills and headache occurred in the CKD-495 75 mg group and were mild or moderate with no significant between-group differences. In addition, 1 patient in the AAF 60 mg group had serum alanine aminotransferase elevation, compared with none of the patients in the CKD-495 75 mg group. Therefore, we conclude that CKD-495 75 mg has a good safety profile in the treatment of acute and chronic gastritis.

*C. cassia* Presl is popular in Chinese medicine and has been used as an antipyretic, antirheumatic, antitumor, antispasmodic, and stomachic treatment.^[19–21]^ Previous studies reported that *C cassia* has antiulcer activity, probably by enhancing defensive factors and by inhibiting the growth of *Helicobacter pylori* and urease activity.^[22–24]^ CKD-495 is a product that is derived from *C cassia* Presl. This new gastric mucoprotective agent mainly depends on enhancing mucosal defensive agents. A previous study reported that *Cinnamomum* not only has an anti-inflammatory effect through the suppression of nitric oxide and prostaglandin E2 production,^[25]^ but also an antioxidative effect by reducing the production of intracellular reactive oxygen species (ROS).^[12,13,25]^

AAF, an ethanol extract of *A asiatica*, has been reported to have anti-inflammatory, antioxidative, and cytoprotective actions in various models of gastric mucosal damage and is currently used in the treatment of gastritis.^[26–30]^ A previous study reported that AAF is effective for gastric mucosal healing, as assessed by endoscopy, in patients with erosive gastritis.^[15]^ The main mechanism of AAF in preventing gastric mucosal injury is likely to involve its antioxidant activity by inhibiting the production of FeSO_4_-induced ROS and preventing H_2_O_2_-induced gastric epithelial damage and anti-inflammatory effects by inhibiting proinflammatory cytokines such as TNF-α.^[30,31]^ Based on the results of these previous studies and phase II trial data, AAF was selected as the control drug for comparison in the current study.

The rationale of CKD-495 for treatment in gastritis is similar to AAF mainly as anti-inflammatory, anti-oxidant activity and secretion of gastric mucus. Despite of similar rationale, the improvement rate of erosion for patients with gastritis of CKD-495 derived from *C cassia* Presl is shown to be better than AAF derived from *A asiatica* in this study. As an in vitro study has indicated that CKD-495 may have a decreasing inflammatory signaling under significant condition compared to AAF^[13]^ and many phytopharmaceutical products possess the medical application through the multitarget activities, it seems that a wide diversity of complex extracts which functions pharmacological effect has caused the difference of clinical outcome, but it needs to be more investigated.

Although CKD-495 was effective in alleviating gastritis, this study has several limitations. Subjects enrolled in this clinical trial were gastritis patients with subjective symptoms and erosions, and evaluation of acute and chronic gastritis was determined by the researcher comprehensively after considering the cause of gastritis, duration of illness, and endoscopic findings. However, among the subjects who were evaluated to have acute gastritis, a small number of subjects with a long disease duration were included because, in this case, it was judged based on the condition of the lesion confirmed by gastroscopy at the time of screening. This is considered as a limitation because the cause of gastritis and *H. pylori* test were not included in the design of the clinical trial. Furthermore, this study was conducted in Korean patients with acute or chronic gastritis, so the generalizability of the findings to other populations requires further study to address any potential ethnic or geographic differences in response to CKD-495 75mg.

In conclusion, this study demonstrated the superiority of CKD-495 75 mg over AAF 60 mg, in terms of the endoscopic improvement rate of erosions, for the treatment of acute and chronic gastritis. This new mucoprotective agent, CKD-495, can be considered the therapy of choice for symptomatic relief and healing of acute or chronic erosive gastritis.

## Acknowledgments

Editorial assistance, including English language editing, was provided by David P. Figgitt PhD, ISMPP CMPP, Content Ed Net, with funding from Chong Kun Dang Pharmaceutical Corp.

## Author contributions

**Investigation:** Soo Teik Lee, Sung Kook Kim, Hoon Jai Chun, Geun Am Song, Dong Ho Lee, Jae Jun Kim, Jin Il Kim, Young Chan Lee, Tae Nyeun Kim, Sam Ryong Jee, Seon-Young Park, Jae Gyu Kim, Jong-Jae Park, Sang Gyun Kim, Jae Myung Park, Jung Ho Park, Oh Young Lee.

**Methodology:** Shin Jung Park.

**Writing – original draft:** Seung Young Seo.

## Supplementary Material


