# Supplementary material for: Efficacy and safety of CKD-495 in acute and chronic gastritis: A Phase III superiority clinical trial
Source: Medicine (Baltimore). 2023 Dec 8;102(49):e35926. doi: 10.1097/MD.0000000000035926 (PMC10713194; doi:10.1097/MD.0000000000035926)
Supplement: Supplementary file 1 [file medi-102-e35926-s001.docx]

**Supplementary Data**

**Table S1**

**Institutional Review Boards and approval codes**

| **Institutional Review Board** | **Approval code** |
| --- | --- |
| Hanyang University College of Medicine | HYUH 2019-11-019 |
| Kyungpook National University School of Medicine | KNUH 2019-11-020 |
| Korea University College of Medicine Anam Hospital | 019AN0495 |
| Pusan National University School of Medicine | 1912-002-098 |
| Seoul National University Bundang Hospital | B-1911-579-003 |
| Samsung Medical Center, Sungkyunkwan University School of Medicine | SMC 2019-11-087 |
| The Catholic University of Korea, Yeouido St. Mary’s Hospital | SC19MDDT0151 |
| Yeungnam University College of Medicine | YUMC 2019-11-019 |
| Inje University Busan Paik Hospital | BPIRB 2019-01-230 |
| Chonnam National University Hospital | CNUH-2019-374 |
| Jeonbuk National University Hospital | CUH 2019-11-005 |
| Severance Hospital, Yonsei University College of Medicine | 4-2019-1053 |
| Chung-Ang University College of Medicine | 1911-026-395 |
| Korea University Guro Hospital | 2020GR0035 |
| Seoul National University College of Medicine | J-2001-042-1093 |
| The Catholic University of Korea, Seoul St. Mary’s Hospital | KC21MDDT0157 |
| Kangbuk Samsung Hospital, Sungkyunkwan University School of Medicine | KBSMC 2021-01-063 |
